# Correlation between Concentrations of Ni and Y in Y-Doped BaZrO_3_ Electrolyte in Co-Sintered Cells: A Case of Controlled NiO Activity by Using MgO-NiO Solid Solution as Anode Substrate

**DOI:** 10.3390/membranes9080095

**Published:** 2019-08-02

**Authors:** Donglin Han, Kenji Kuno, Tetsuya Uda

**Affiliations:** Department of Materials Science and Engineering, Kyoto University, Yoshida Honmachi, Sakyo-ku, Kyoto 606-8501, Japan

**Keywords:** proton conductor, barium zirconate, fuel cell, Ni diffusion, magnesia

## Abstract

BaZr_0.8_Y_0.2_O_3-δ_ (BZY20) is promising to be applied as an electrolyte in fuel cells, electrolysis cells, etc. However, when a half cell composed of a BZY20 electrolyte layer and a BZY20-NiO composite anode substrate is co-sintered (1400–1600 °C), Ni diffuses from the anode substrate into the electrolyte layer. Y content in the electrolyte layer decreases dramatically, since BZY20 cannot be equilibrated with NiO at such high temperature. Such Ni diffusion and Y loss are detrimental to the electrochemical performance of the electrolyte layer. In this work, we added MgO-NiO solid solution into the anode substrate to adjust the NiO activity (*a*_NiO_) during the co-sintering process, and used three different co-sintering methods to control the BaO activity (*a*_BaO_). The results revealed that by decreasing *a*_NiO_ in the system, the as-co-sintered electrolyte layer had the composition shifting towards the direction of high Y and low Ni cation ratios. A clear correlation between the intra-grain concentration of Ni and Y was confirmed. In other words, to prepare the electrolyte with the same Y cation ratio, the Ni diffusion into the electrolyte layer can be suppressed by using the MgO-NiO solid solution with a high MgO ratio and a low Ni ratio. Moreover, by increasing *a*_BaO_, we found that the Y cation ratio increased and approached the nominal value of the pristine BZY20, when Mg_1−*x*_Ni*_x_*O (*x* = 0.3 and 0.5) was used. In summary, both *a*_NiO_ and *a*_BaO_ play important roles in governing the composition of the electrolyte layer prepared by the co-sintering process. To evaluate the quality of the electrolyte layer, both the intra-grain Y and Ni concentrations should be carefully checked.

## 1. Introduction

A fuel cell can be regarded as an assembling of cathode, electrolyte and anode layers. The electrolyte is the most important component and needs high ionic conductivity of at least 0.01 Scm^−1^ [1]. A total of 20 mol % Y-doped BaZrO_3_ (BaZr_0.8_Y_0.2_O_3−δ_ (BZY20)) shows proton conductivity of about 0.01 Scm^−1^ even at 450 °C [2,3,4] and excellent chemical stability against the reaction with water vapor and carbon dioxide [5,6,7]. BZY20 is therefore one of the most promising electrolyte candidates for intermediate temperature fuel cells (IT-FCs). To minimize ohmic resistance from the electrolyte, one ordinary strategy is to construct an anode-supported structure. The electrolyte layer is supported mechanically by the anode layer, thereby can be thinned to several tens of micrometers. However, the cell fabrication process faces a severe challenge; during the co-sintering process performed at 1400–1600 °C, nickel diffuses from the anode substrate (a mixture of BZY20 and NiO) into the BZY20 electrolyte [8,9]. Furthermore, the Y content decreases dramatically due to formation of some second phases like BaY_2_NiO_5_ [10]. For example, we found that in the cell co-sintered at 1500 °C, about 1–1.5% (cation ratio) Ni existed in the BZY20 electrolyte [8,9], and the cation ratio of Y decreased to about 5% [9] (the Y content in the pristine BZY20 is 10%). The proton conductivity of Y-doped BaZrO_3_ suffers from both the Ni diffusion [8,11,12,13] and the loss of Y [4,14]. Moreover, the diffusion of Ni also lowers the transport number of proton conduction in an oxidizing atmosphere [8,13]. Due to the aforementioned reasons, the appropriateness of such process is questionable, when it is utilized to fabricate the BZY20 electrolyte-based cells with the anode substrate containing NiO (lately reduced to Ni metal), in other words, under the condition of the NiO activity of unity (*a*_NiO_ = 1).

Naturally, one may wonder whether the diffusion of nickel into the electrolyte can be suppressed if *a*_NiO_ is lowered. MgO and NiO are both of a cubic fluorite structure ($Fm\bar{3}m$), and form a miscible solid solution over the full composition range [15]. It is thus easy to adjust *a*_NiO_ by applying the MgO-NiO solid solution with controlled composition in the anode substrate. The attempt to apply the MgO-NiO solid solution in solid oxide fuel cells (SOFCs) has already been reported, but is not with the purpose to control *a*_NiO_ [16].

In this work, the solid solution of Mg_1−*x*_Ni*_x_*O (*x* = 0.3, 0.5, 0.7) was prepared and added into the anode substrate. After spin-coating the BaZr_0.8_Y_0.2_O_3−δ_ (BZY20) electrolyte layers onto the anode substrates, the half cells were subjected subsequently to the co-sintering process performed at 1600 °C. The phases and the composition in the electrolyte layers in the as-co-sintered half cells were analyzed carefully. Through this work, we propose a strategy to control *a*_NiO_, and thereby suppress the Ni diffusion during the co-sintering process.

## 2. Experimental Methods

### 2.1. Material Preparation

BaZrO_3_ and BaZr_1−*x*_Y*_x_*O_3−δ_ (*x* = 0.02, 0.05, 0.1, 0.12, 0.15, 0.17, 0.2, 0.22) samples were synthesized with the solid-state reaction method. The detailed process was reported in our previous works [17,18]. For convenience, the BaZr_1−*x*_Y*_x_*O_3−δ_ samples were named as BZY100*x*. For example, BZY20 for BaZr_0.8_Y_0.2_O_3−δ_. The as-synthesized powder was pelletized at 392 MPa and heated at 1600 °C for 24 h in artificial oxygen with subsequent pulverization by ball-milling for 50 h to prepare the powders with improved compositional homogeneity [19]. Then, a part of the samples was added with 2 wt % NiO (Nikko Rica Corporation, 99.9%, Tokyo, Japan). The nominal cation ratio of Ni in all these samples was approximately 3.6%. After ball-milling for 10 h for mixing, the mixtures were pelletized at 392 MPa, and embedded in the sacrificial powder with the same composition of the pellets (BZY 100*x*-2 wt % NiO). Finally, the pellets were subjected to sintering at 1500 °C in oxygen for 10 h.

The Mg_1−*x*_Ni*_x_*O (*x* = 0.3, 0.5, 0.7) solid solution was also prepared by the solid-state reaction method. Starting materials of MgO (Wako Pure Chemical Industries, Ltd., 99.9%, Osaka, Japan) and NiO were mixed at desired ratio and ball-milled for 10 h. After being heated at 1000 °C for 10 h, the samples were ball-milled for 10 h to pulverize. Then, the mixtures were pelletized at 14 MPa and heat-treated at 1300 °C for 10 h for synthesizing. The as-synthesized samples were subsequently ball-milled for 50 h to pulverize. Single phases (the powder XRD patterns are shown in supplementary Figures S1 and S2) with average grain size around 1 μm (the EPMA back scattering electron images are shown in Figure S3) were confirmed for the samples after ball-milling.

### 2.2. Half Cell Fabrication

The as-synthesized Mg_1−*x*_Ni*_x_*O (*x* = 0.3, 0.5, 0.7) was mixed with 50 wt % BZY20 by ball-milling for 10 h without adding pore former. The mixtures were pressed at 392 MPa into pellet-like anode substrates with the diameter and thickness of 11 mm and 1 mm, respectively. The BZY20 electrolyte was prepared by spin-coating slurry composed of 50 wt % BZY20, 2 wt % ethylcellulose and 48 wt % α-terpineol onto the surface of the anode substrates using an MS-A100 Spin Coater (Mikasa Co., Ltd., Tokyo, Japan) [19]. Then, the half cells were placed on magnesia crucibles and subjected to co-sintering. For comparison, the half cells composed of BZY20 electrolyte layers and BZY20–60 wt % NiO anode substrate layers were also prepared following the same approach.

**Figure 1:**
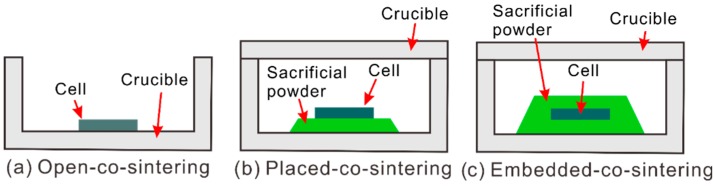
Schematics of (**a**) open-co-sintering, (**b**) placed-co-sintering, and (**c**) embedded-co-sintering methods used in this work. MgO crucibles were used in the experiments. For the placed-co-sintering and embedded-co-sintering methods, the half cells were placed on and embedded in the sacrificial powder of BZY20-1 wt % BaCO_3_, respectively.

Since BaO is easy to evaporate at high temperature, which results in Ba-deficiency and segregation of Y_2_O_3_ [20], in this work, three methods were applied to vary the BaO activity (*a*_BaO_) in atmosphere during the co-sintering process. Figure 1a shows the case without using any sacrificial powder. Figure 1b,c shows the ones using sacrificial powder with the half cells being placed on and embedded in the sacrificial powder of BZY20-1 wt % BaCO_3_, respectively. For convenience, these three methods are named as open-co-sintering, placed-co-sintering, and embedded-co-sintering methods, respectively. *a*_BaO_ is the lowest in the open-co-sintering method, and the highest in the embedded-co-sintering method.

A temperature of 1600 °C was selected as the co-sintering temperature in this work. Through a preliminary experiment on the half cells composed of the BZY20 electrolyte layers and the BZY20-50 wt % Mg_0.3_Ni_0.7_O anode substrate layers, the electrolyte is porous after co-sintering at 1500 °C (Figure S4a), but turned to be dense after co-sintering at 1600 °C (Figure S4b).

### 2.3. Characterization

X-ray diffraction (XRD) measurements were performed using Cu *K*α radiation with X’Pert PRO MPD (PANalytical, Almelo, The Netherlands). Microstructures were observed by an electron probe microanalyzer (EPMA) with JXA-8530F (JEOL, Tokyo, Japan), and local composition was measured with wavelength-dispersive X-ray spectroscopy (EPMA-WDS). Average composition was determined by inductively coupled plasma atomic emission spectroscopy (ICP-AES) with SPS4000 (Seiko Instruments Inc., Chiba, Japan). Before the EPMA observation, the half cells were fixed in resin, and a fractured cross-section was polished by JEOL IB-19510CP Cross Section Polisher.

## 3. Results

### 3.1. Co-Sintered Half Cells

#### 3.1.1. Morphology of Cross-Section Area

**Figure 2:**
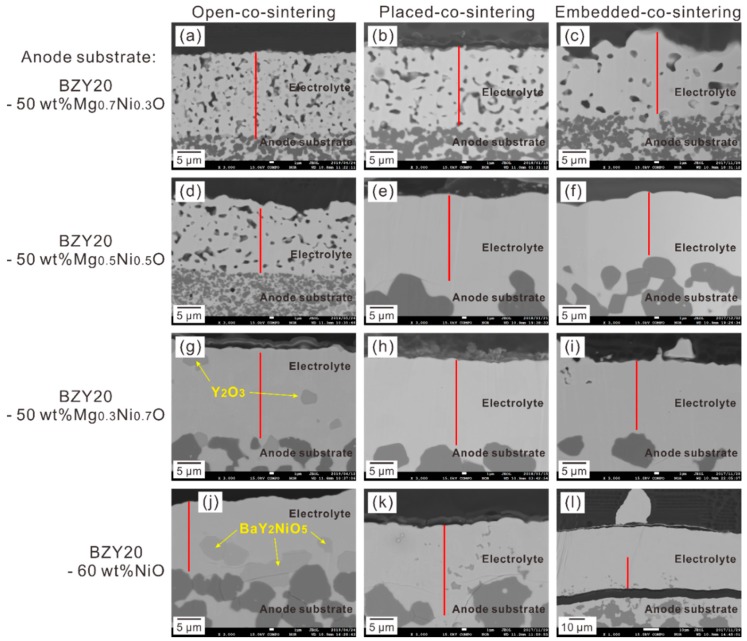
Back-scattering electron images of the cross-section area of the half cells with the BZY20-50 wt % Mg_0.7_Ni_0.3_O anode substrate prepared with the methods of (**a**) open-co-sintering, (**b**) placed-co-sintering, (**c**) embedded-co-sintering, and the BZY20-50 wt% Mg_0.5_Ni_0.5_O anode substrate prepared with the methods of (**d**) open-co-sintering, (**e**) placed-co-sintering, (**f**) embedded-co-sintering, and the BZY20-50 wt% Mg_0.3_Ni_0.7_O anode substrate prepared with the methods of (**g**) open-co-sintering, (**h**) placed-co-sintering, (**i**) embedded-co-sintering, and the BZY20-60 wt % NiO anode substrate prepared with the methods of (**j**) open-co-sintering, (**k**) placed-co-sintering, (**l**) embedded-co-sintering. All the half cells were co-sintered at 1600 °C in oxygen for 10 h with the methods shown in Figure 1. The red line indicates the position for EPMA-WDS line analysis. It should be noted that the scale bar of (**l**) is larger than those of the other images.

The back-scattering electron (BSE) images of the cross-section area of the half cells after co-sintering at 1600 °C are shown in Figure 2. The morphology of the electrolyte layer appears to be dependent on both the components of the anode substrates and the co-sintering methods, which determine *a*_NiO_ and *a*_BaO_, respectively.

For those using Mg_0.7_Ni_0.3_O in the anode substrate, the electrolyte layer is porous in morphology, regardless of the co-sintering methods. However, it is clear to see that the porosity of the electrolyte decreases when the half cells were placed on or embedded in the BZY20-1 wt % BaCO_3_ sacrificial powder (Figure 2b,c). When Mg_0.5_Ni_0.5_O was added in the anode substrate, the electrolyte layer is porous by applying the open-co-sintering method (Figure 2d), but becomes dense by co-sintering with the sacrificial powder (Figure 2e,f). Since 1600 °C is sufficient for the densification and grain growth of Y-doped BaZrO_3_ (BZY) [2,4], one possible reason for such porous morphology of the electrolyte layer under the relatively low *a*_NiO_ condition is due to the smaller shrinkage ratio of the anode substrate than that of the electrolyte layer. Therefore, the densification of the electrolyte layer is inhibited by the tensional stress raised from the interface with the anode substrate. These results indicate the importance to control the sintering behaviour of the anode substrate. By increasing *a*_BaO_, some second phases, e.g., BaY_2_NiO_5_ and BaNiO_3−δ_, form during heating up to 1600 °C. These second phases are reported to be effective on improving the sinterability of BZY [21,22]. Furthermore, from the BaO-ZrO_2_-YO_1.5_ pseudoternary phase diagram [23], the Ba-rich composition induces the generation of liquid phases, which also possibly promote the densification of the electrolyte layer. Notably, some locally Ba-rich area forms (for example, at the grain boundary) as the result of the diffusion of Ni into the intra-grain with BaO excluded [11].

With regard to the half cells containing Mg_0.3_Ni_0.7_O and NiO in the anode substrate, dense electrolyte was obtained, regardless of which co-sintering method was used. Notably, for the half cell with the BZY20-60 wt % NiO anode substrate, which was co-sintered by embedding in the BZY20-1 wt % BaCO_3_ sacrificial powder, the sacrificial powder adhered firmly on the surface of the electrolyte layer and cannot be removed (Figure S5b). The thickness of the electrolyte layer became 100 μm (Figure 2l), greatly thicker than around 20 μm in the other cases. One possible reason might be that some sacrificial powder was allowed to form, thereby thicken the electrolyte layer due to the improved sinterability of BZY under the high *a*_NiO_ and *a*_BaO_ condition. We also see some trace possibly belonging to the liquid phase in the electrolyte layer in the half cells using the BZY20-60 wt % NiO anode substrate, which were co-sintered with the sacrificial powder (Figure 2k,l).

#### 3.1.2. Phase Purity in Electrolyte

From the back-scattering electron (BSE) image, phases with different composition can be distinguished due to the difference in contrast, which is dependent on the average atomic number. For the half cells prepared by the open-co-sintering method, it is clear that second phases formed in the electrolyte layer of the half cells containing Mg_0.3_Ni_0.7_O (Figure 2g) and NiO (Figure 2j) in the anode substrate. Through the EPMA-WDS elemental mapping analysis, these second phases were identified to be Y_2_O_3_ (Figure S12) and BaY_2_NiO_5_ (Figure S13), respectively. Interfering by the pores, it is difficult to confirm the phase purity of the electrolyte of the half cells containing Mg_0.7_Ni_0.3_O and Mg_0.5_Ni_0.5_O from the BSE images. However, the results of the elemental mapping analysis (Figures S10 and S11) indicate the segregation of Y_2_O_3_, in agreement with the results of the XRD analysis (Figures S6 and S7). For the half cells applying Mg_0.5_Ni_0.5_O and Mg_0.3_Ni_0.7_O in the anode substrate, which were co-sintered by placing on or embedding in the BZY20-1 wt % BaCO_3_ sacrificial powder, we can hardly see any second phase from the BSE images of the cross-section area. But as mentioned, there is some trace possibly belonging to the liquid phase existing in the electrolyte layer of the cell containing NiO in the anode substrate (Figure 2k,l).

#### 3.1.3. EPMA-WDS Line Analysis

**Figure 3:**
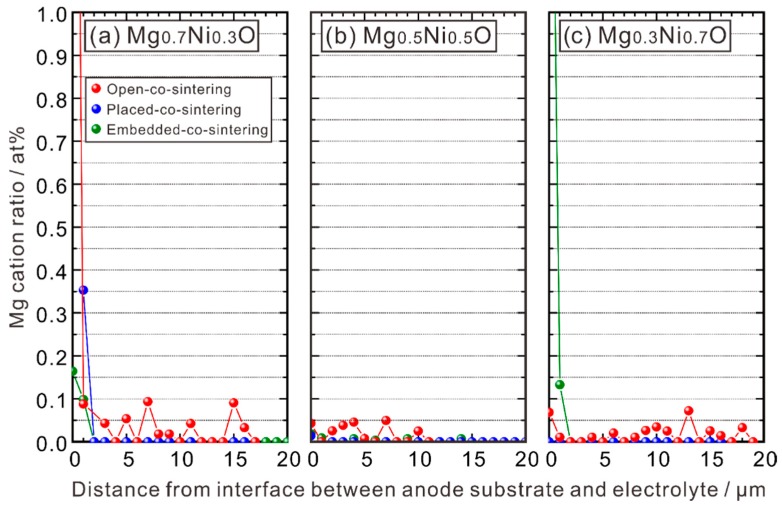
Mg cation ratio in the electrolyte layer of the half cells with the anode substrates of (**a**) BZY20-50 wt % Mg_0.7_Ni_0.3_O, (**b**) BZY20-50 wt % Mg_0.5_Ni_0.5_O, and (**c**) BZY20-50 wt % Mg_0.3_Ni_0.7_O. All the half cells were co-sintered at 1600 °C in oxygen for 10 h with the method shown in Figure 1. The Mg cation ratio was measured with EPMA-WDS line analysis following the position shown in Figure 2 (red line) from the interface between the anode substrate and the electrolyte.

**Figure 4:**
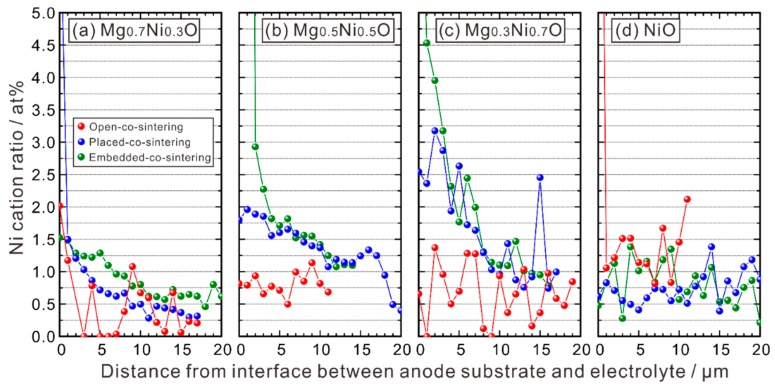
Ni cation ratio in the electrolyte layer of the half cells with the anode substrates of (**a**) BZY20-50 wt % Mg_0.7_Ni_0.3_O, (**b**) BZY20-50 wt % Mg_0.5_Ni_0.5_O, (**c**) BZY20-50 wt % Mg_0.3_Ni_0.7_O, and (**d**) BZY20-50 wt % NiO. All the half cells were co-sintered at 1600 °C in oxygen for 10 h with the method shown in Figure 1. The Ni cation ratio was measured with EPMA-WDS line analysis following the position shown in Figure 2 (red line) from the interface between the anode substrate and the electrolyte.

EPMA-WDS line analysis was performed along the red lines shown in Figure 2 to measure the composition across the electrolyte layer. The cation ratio of Mg is shown in Figure 3, in which the abscissa is the distance from the interface between the electrolyte layer and the anode substrate towards the surface of the electrolyte layer. One can see that Mg is hardly detectable, and its concentration is close to noise level. These results indicate that the MgO-NiO solid solution is appropriate to adjust *a*_NiO_ without introducing any impact on the basic properties of BZY from MgO. In contrast, existence of Ni is clearly confirmed in the electrolyte, as shown in Figure 4.

**Figure 5:**
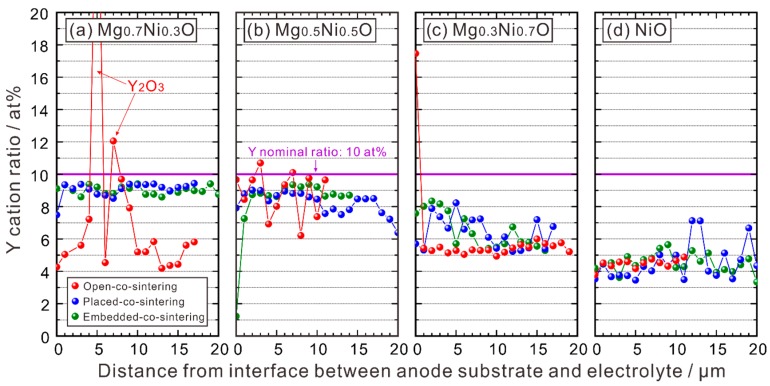
Y cation ratio in the electrolyte layer of the half cells with the anode substrates of (**a**) BZY20-50 wt % Mg_0.7_Ni_0.3_O, (**b**) BZY20-50 wt % Mg_0.5_Ni_0.5_O, (**c**) BZY20-50 wt % Mg_0.3_Ni_0.7_O, and (**d**) BZY20-50 wt % NiO. All the half cells were co-sintered at 1600 °C in oxygen for 10 h with the method shown in Figure 1. The Y cation ratio was measured with EPMA-WDS line analysis following the position shown in Figure 2 (red line) from the interface between the anode substrate and the electrolyte.

The Y concentration is shown in Figure 5. For the half cells using NiO in the anode substrate, the Y cation ratio in the electrolyte is about 4–5%, and there is no obvious difference in the Y content when different co-sintering methods are used. For the half cells which use Mg_0.7_Ni_0.3_O and Mg_0.5_Ni_0.5_O in the anode substrate and were co-sintered without the sacrificial powder, the Y cation ratio fluctuates greatly, due to the existence of pores and segregation of Y_2_O_3_ in the electrolyte layer. However, compared with the case using NiO, one can see that, in general, for the half cells which use the MgO-NiO solid solution in the anode substrate, the Y cation ratio increases when the BZY20-1 wt % BaCO_3_ sacrificial powder was used. The results of Ba and Zr cation ratios are given in the Supplementary Materials.

#### 3.1.4. Relationship between Ni and Y Concentrations

**Figure 6:**
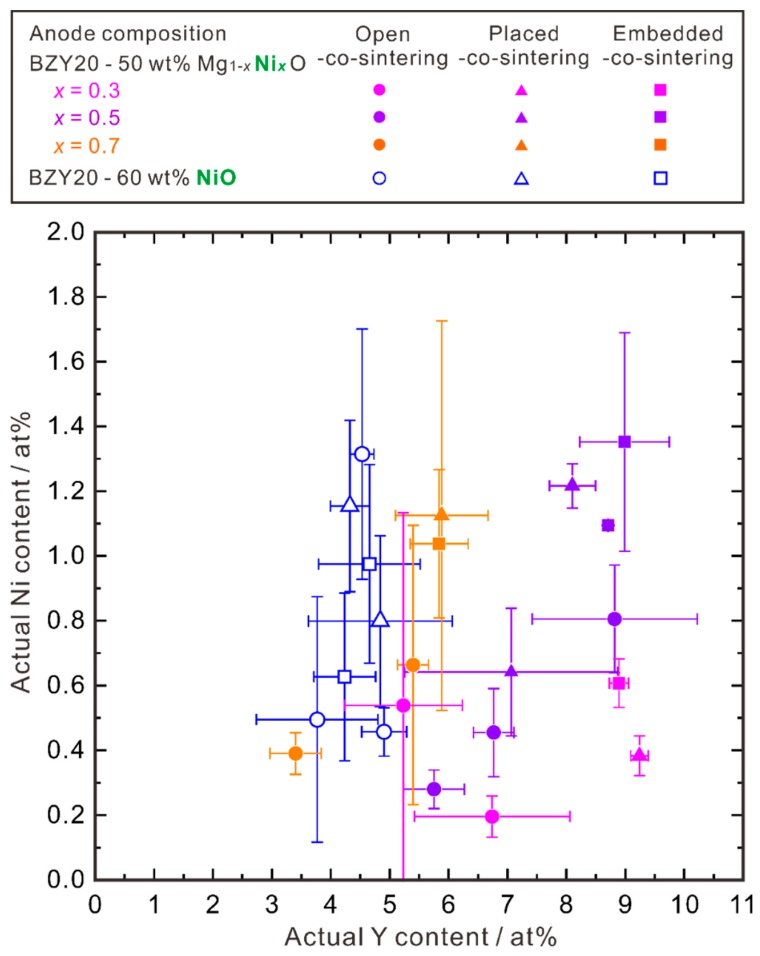
Relationship between the Y and Ni content in the electrolyte layer of the half cells with the anode substrate containing 60 wt % NiO, or 50 wt % Mg_1−*x*_Ni*_x_*O (*x* = 0.3, 0.5, 0.7). The half cells were co-sintered at 1600 °C in O_2_ for 10 h. Three different co-sintering strategies as shown in Figure 1 were adopted to control the activity of BaO during co-sintering. The cation ratio of Ni and Y were determined by EPMA-WDS point analysis collected in the area of about 12 to 20 μm apart from the interface between the electrolyte and anode substrate. The points apparently belonging to the second phases (NiO, Y_2_O_3_, etc.) were excluded. The standard deviation (σ) was used for drawing the error bars.

Our initial target of using the MgO-NiO solid solution is to suppress the diffusion of Ni by lowering *a*_NiO_. From Figure 4, we can see that for the half cells prepared with the placed-co-sintering and embedded-co-sintering methods, the Ni cation ratio in the electrolyte layer decreases with the increasing MgO ratio in the Mg_1−*x*_Ni*_x_*O (*x* = 0.3, 0.5, 0.7). However, although the half cells using the NiO-contained anode substrate is expected to show the highest Ni cation ratio in the electrolyte layer, we found that the Ni cation ratio is low, comparable to the case using the Mg_0.7_Ni_0.3_O-contained anode substrate. Noticing that the Y content decreases with the increasing NiO ratio in the anode substrate, we supposed that there might be a certain relationship between the cation ratios of Ni and Y, and plotted it in Figure 6. Here, the average cation ratios of Ni and Y were calculated by using the data collected by EPMA-WDS line analysis in the area of about 12 to 20 μm apart from the interface between the electrolyte and anode substrate. The points apparently belonging to the second phases (NiO, Y_2_O_3_, etc.) were excluded.

Some samples with the same cell structure and the same co-sintering method were reproduced several times, but we have to say that the reproducibility is not very good at present. However, we can see a very clear general tendency; that is, with the increasing MgO ratio in the MgO-NiO solid solution, the composition of the electrolyte layer after co-sintering shifts towards the direction of high Y and low Ni cation ratios. In other words, for the electrolyte layer with the same Y content, the diffusion of Ni into the electrolyte can be suppressed by lowering *a*_NiO_, namely, by increasing the MgO ratio in the MgO-NiO solid solution.

So, the cation ratio of Ni diffused into the BZY electrolyte layer is not an independent parameter and appears to correlate with the Y cation ratio. The oxide ion vacancies induced by the Y cations possibly increases the Ni content.

### 3.2. BaZr_1−x_Y_x_O_3−δ_ (x = 0–0.22)-2 wt % NiO Samples

**Figure 7:**
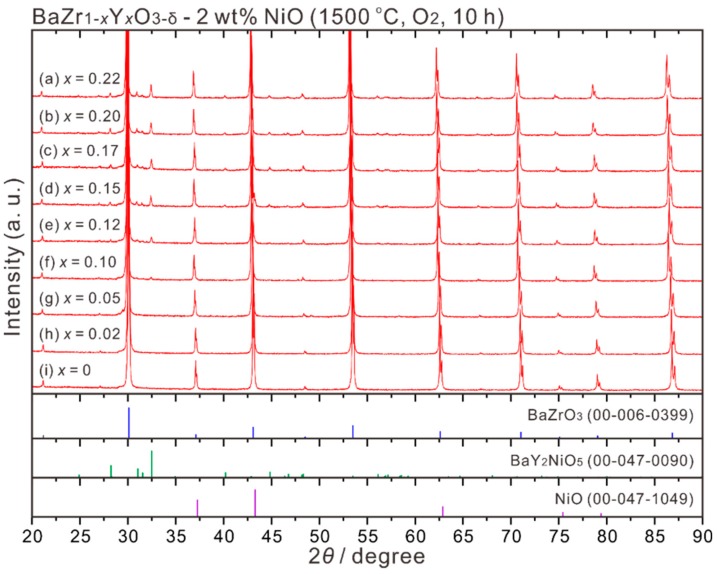
Powder XRD patterns of the BaZr_1−*x*_Y*_x_*O_3−δ_-2 wt % NiO (*x* = 0, 0.02, 0.05, 0.1, 0.12, 0.15, 0.17, 0.20, 0.22) samples, which were sintered at 1500 °C in oxygen for 10 h.

To further understand the correlation between the cation ratios of Ni and Y in BZY, bulk samples of BaZr_1−*x*_Y*_x_*O_3−δ_ (*x* = 0, 0.02, 0.05, 0.1, 0.12, 015, 0.17, 0.2, 0.22) added with 2 wt % NiO were prepared by sintering at 1500 °C in an oxygen atmosphere for 10 h. The powder XRD patterns shown in Figure 7 indicate that for BaZrO_3_ and BaZr_1−*x*_Y*_x_*O_3−δ_ (*x* = 0.02, 0.05) mixed with 2 wt % NiO, almost only the peaks belonging to the perovskite phase appear (there is a weak peak at around 27°, unable to be identified). However, for the BZY-2 wt % NiO samples containing Y content higher than 0.1, diffraction peaks belonging to BaY_2_NiO_5_ appear. These results deviate from the conclusion that BaZr_1−*x*_Y*_x_*O_3−δ_ (*x* ≤ 0.12) can achieve a two-phase equilibrium with NiO at 1500 °C, which is reported in our recent study on the phase diagram of BaO-ZrO_2_-YO_1.5_-NiO [10]. The BaY_2_NiO_5_ phase might form during cooling at the rate about 3.85 °C min^−1^ from 1500 °C to room temperature, while in our previous phase diagram study [10], the samples were finally quenched.

**Figure 8:**
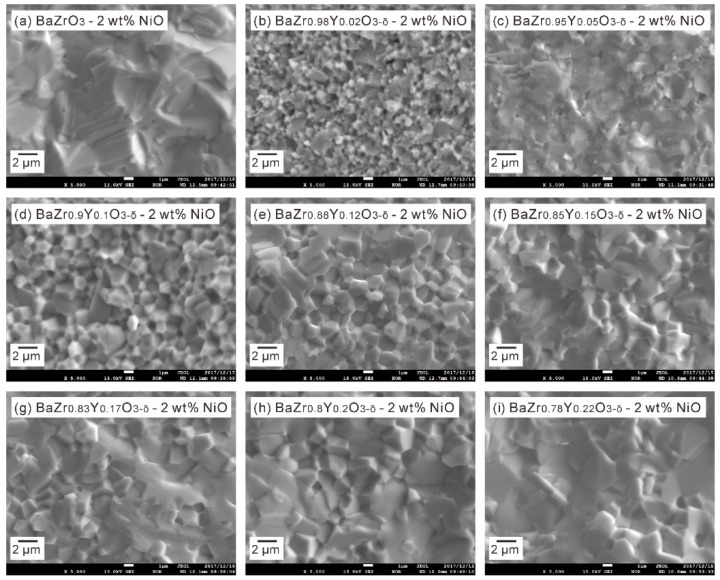
Second electron images of the BaZr_1−*x*_Y*_x_*O_3−δ_-2 wt % NiO (*x* = 0, 0.02, 0.05, 0.1, 0.12, 0.15, 0.17, 0.20, 0.22) samples, which were sintered at 1500 °C in oxygen for 10 h.

We reported previously that the BZY samples with the Y content of 0.5, 0.1, 0.12 and 0.15 showed a bimodal microstructure after sintering at 1600 °C for 24 h [4,14]. However, as shown in Figure 8, all the samples exhibit a microstructure with a uniform grain size, attributing to the role of NiO as the sintering additive for BZY [21,22].

**Figure 9:**
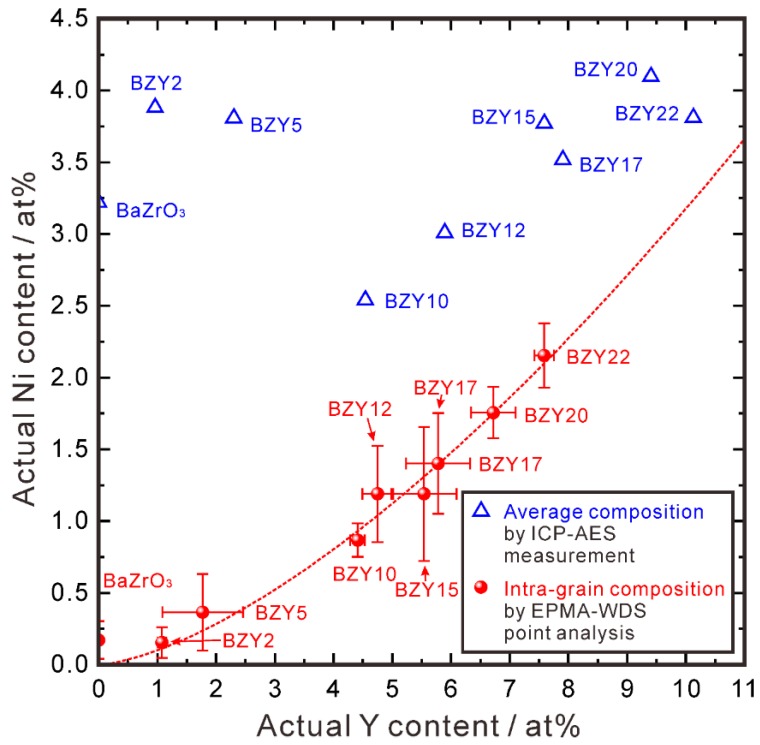
Relationship between the Y and Ni content in the samples with the nominal composition of BaZr_1−*x*_Y*_x_*O_3−δ_-2 wt % NiO (*x* = 0, 0.02, 0.05, 0.1, 0.12, 0.15, 0.17, 0.20, 0.22) samples, which were sintered at 1500 °C in oxygen for 10 h. Both the average compositions determined by ICP-AES measurement, and those of intra-grain determined by EPMA-WDS point analysis were plotted. In the figure, the nominal composition of BaZr_1−*x*_Y*_x_*O_3−δ_ were expressed as BZY(100 × *x*) in short. The standard deviation (*σ*) was used for drawing the error bars. To verify the reliability of the difference in the composition determined by EPMA-WDS and ICP-AES analyses, EPMA-WDS area analyses were performed to measure the composition of four different square areas (100 μm × 100 μm) of BZY12-2 wt % NiO. The average cation ratio of Y and Ni is 5.89% and 2.69%, respectively, very close to 5.90% and 3.00%, respectively.

Since the grain size of all the samples is not smaller than 1 μm, EPMA-WDS point analysis was applied to determine the intra-grain composition, and the results are plotted in Figure 9 (red ball symbols). Interestingly, one can clearly see that the Ni content increases with the Y content, indicating that the Ni and Y cation ratio correlate in the intra-grain area of BZY. Furthermore, comparing the intra-grain composition and average composition (measured by ICP-AES analysis), the intra-grain Ni content is smaller than the average Ni content for all the samples. For BaZr_1−*x*_Y*_x_*O_3−δ_ (*x* ≥ 0.1), the intra-grain Y content is smaller than the average value (measured by ICP-AES analysis), with the excessive Y and Ni accommodated in the second phase of BaY_2_NiO_5_ (an example of WDS elemental mapping of BaZr_0.78_Y_0.22_O_3−δ_-2 wt % NiO is given in Figure S16). Whereas for BaZr_1−*x*_Y*_x_*O_3−δ_ (*x* ≤ 0.05), the intra-grain Y content is close to the average one, and only NiO was found to remain (an example of WDS elemental mapping of BaZrO_3_-2 wt % NiO is given in Figure S17). Since the diffraction peaks of NiO is very close to that of BZY (Figure 7), we cannot confirm the existence of NiO from the XRD patterns.

## 4. Discussion

Ideally, the electrolyte should maintain a single phase without any compositional change after the co-sintering process, to fully activate its electrochemical properties. However, it seems to be difficult for BaZr_0.8_Y_0.2_O_3−δ_ (BZY20). Firstly, a two-phase equilibrium cannot be achieved between BZY20 and NiO at 1500 °C [10]. The BaY_2_NiO_5_ second phase forms, accompanied with the decrease in Y content from 10% (the pristine BZY20) to about 6% [10]. Secondly, the high temperature for co-sintering accelerates the evaporation of BaO [20] to leave from the surface of the electrolyte layer into the atmosphere. Thereby, it is easy for the electrolyte layer to be Ba-deficient. When the electrolyte loses too much BaO, Y_2_O_3_ segregates, resulting in the decrease of Y content in the electrolyte layer [24].

For the half cells using BZY20-60 wt % NiO as the anode substrate, the Y content decreases dramatically from 10% to 4–5% after co-sintering at 1600 °C. And there is no obvious change in the Y content when the co-sintering methods is changed. However, for the half cells using Mg_0.7_Ni_0.3_O and Mg_0.5_Ni_0.5_O in the anode substrate, the Y content increases obviously when the sacrificial powder was used during the co-sintering process. Furthermore, from the results of the XRD and EPMA-WDS elemental mapping analyses, BaY_2_NiO_5_ and Y_2_O_3_ were found in the electrolyte layer in the cells using NiO and MgO-NiO solid solution in the anode substrate, respectively, after co-sintering without the sacrificial powder. These results can be explained by the activities of NiO and BaO. In the case of *a*_NiO_ = 1, according to the phase equilibrium of the BaO-ZrO_2_-YO_1.5_-NiO system, BaY_2_NiO_5_ forms, and Y content in the BZY phase decreases. However, when *a*_NiO_ was lowered sufficiently, for example, to 0.5 or 0.3, BaY_2_NiO_5_ cannot form. The two-phase equilibrium between BZY20 and Mg_1−*x*_Ni*_x_*O (*x* ≤ 0.5) thereby turns to be possible. In this case, the decrease in the Y content resulted from the evaporation of BaO, and the Y content can thereby be increased by keeping high *a*_BaO_.

## 5. Conclusions

In this work, MgO-NiO solid solution was added into the anode substrate to adjust the NiO activity during the co-sintering process. In addition, three different co-sintering methods were applied to control the BaO activity. The results revealed that with the decreasing NiO ratio and increasing MgO ratio in the MgO-NiO solid solution, the composition of the electrolyte layer after co-sintering shifted towards the direction of high Y and low Ni cation ratios. Furthermore, we prepared a series of as-sintered samples of BZY-2 wt % NiO with different Y content in BZY and confirmed a clear correlation between the intra-grain Ni and Y cation ratios. The Ni content in intra-grain increases with the Y content. Comparing the electrolyte with the same Y cation ratio, the Ni diffusion into the electrolyte layer can be suppressed by using the MgO-NiO solid solution with a high MgO ratio and a low Ni ratio. Moreover, when Mg_1−*x*_Ni*_x_*O (*x* = 0.3 and 0.5) was used, increasing *a*_BaO_ during the co-sintering process resulted in an increase of the Y cation ratio, approaching the nominal value of pristine BZY20. Both *a*_NiO_ and *a*_BaO_ play important roles in governing the composition of the electrolyte layer prepared by the co-sintering process.

## Supplementary Materials

The following are available online at https://www.mdpi.com/2077-0375/9/8/95/s1, Figure S1: Powder XRD patterns of as-synthesized MgO-NiO solid solution samples; Figure S2: (a) Shifting of (220) diffraction peak towards low angle side, and (b) increasing lattice constant of the MgO-NiO solid solution samples with the increasing Mg concentration [25]; Figure S3: Back scattering electron images of the powders of as-synthesized MgO-NiO solid solution samples; Figure S4: Back scattering electron images of the half cells composed of a BZY20 electrolyte layer and a BZY20-50 wt % Mg_0.3_Ni_0.7_O anode substrate with the open-co-sintering method; Figure S5: Optical images of the half cells composed of the BZY20 electrolyte layer and (a) BZY20-50 wt % Mg_0.3_Ni_0.7_O, and (b) BZY20-60 wt % NiO anode substrates after co-sintering at 1600 °C; Figure S6: XRD patterns of the electolyte side on the half cells composed of BZY20 electrolyte layers and BZY20-50 wt % Mg_0.7_Ni_0.3_O anode substrate co-sintered at 1600 °C; Figure S7: XRD patterns of the electolyte side on the half cells composed of BZY20 electrolyte layers and BZY20-50 wt % Mg_0.5_Ni_0.5_O anode substrate co-sintered at 1600 °C; Figure S8: XRD patterns of the electolyte side on the half cells composed of BZY20 electrolyte layers and BZY20-50 wt % Mg_0.3_Ni_0.7_O anode substrate co-sintered at 1600 °C: Figure S9: XRD patterns of the electolyte side on the half cells composed of BZY20 electrolyte layers and BZY20-60 wt % NiO anode substrate co-sintered at 1600 °C; Figure S10: EPMA-WDS elemental mapping of the cross-section area of the half cell composed of a BZY20 electrolyte and a BZY20-50 wt % Mg_0.7_Ni_0.3_O anode substrate using the open-co-sintering method; Figure S11: EPMA-WDS elemental mapping of the cross-section area of the half cell composed of a BZY20 electrolyte and a BZY20-50 wt % Mg_0.5_Ni_0.5_O anode substrate; Figure S12: EPMA-WDS elemental mapping of the cross-section area of the half cell composed of a BZY20 electrolyte and a BZY20-50 wt % Mg_0.3_Ni_0.7_O anode substrate; Figure S13: EPMA-WDS elemental mapping of the cross-section area of the half cell composed of a BZY20 electrolyte and a BZY20-60 wt % NiO anode substrate; Figure S14: Ba cation ratio in the electrolyte layer of the half cells with the anode substrates of (a) BZY20-50 wt % Mg_0.7_Ni_0.3_O, (b) BZY20-50 wt % Mg_0.5_Ni_0.5_O, (c) BZY20-50 wt % Mg_0.3_Ni_0.7_O, and (d) BZY20-50 wt % NiO.; Figure S15: Zr cation ratio in the electrolyte layer of the half cells with the anode substrates of (a) BZY20-50 wt % Mg_0.7_Ni_0.3_O, (b) BZY20-50 wt % Mg_0.5_Ni_0.5_O, (c) BZY20-50 wt % Mg_0.3_Ni_0.7_O, and (d) BZY20-50 wt % NiO.; Figure S16: EPMA-WDS elemental mapping of the cross-section of the BaZr_0.78_Y_0.22_O_3−δ_-2 wt % NiO pellet after sintering at 1500 °C; Figure S17: EPMA-WDS elemental mapping of the cross-section of the BaZrO_3_-2 wt % NiO pellet after sintering at 1500 °C.
