# Supplementary material for: Correlation between Concentrations of Ni and Y in Y-Doped BaZrO3 Electrolyte in Co-Sintered Cells: A Case of Controlled NiO Activity by Using MgO-NiO Solid Solution as Anode Substrate
Source: Membranes (Basel). 2019 Aug 2;9(8):95. doi: 10.3390/membranes9080095 (PMC6723951; doi:10.3390/membranes9080095)
Supplement: Supplementary file 1 [file membranes-09-00095-s001.pdf]

membranes

Supplementary Materials: Correlation between Concentrations of Ni and Y in Y-doped $BaZrO_3$ Electrolyte in Co-sintered Cells: A Case of Controlled NiO Activity by Using MgO-NiO Solid Solution as Anode Substrate

Donglin Han *, Kenji Kuno and Tetsuya Uda *

Department of Materials Science and Engineering, Kyoto University, Yoshida Honmachi, Sakyo-ku, Kyoto 606-8501, Japan

* Correspondence: han.donglin.8n@kyoto-u.ac.jp (D.H.); uda_lab@aqua.mtl.kyoto-u.ac.jp (T.U.); Tel.: +81-75-753-5445

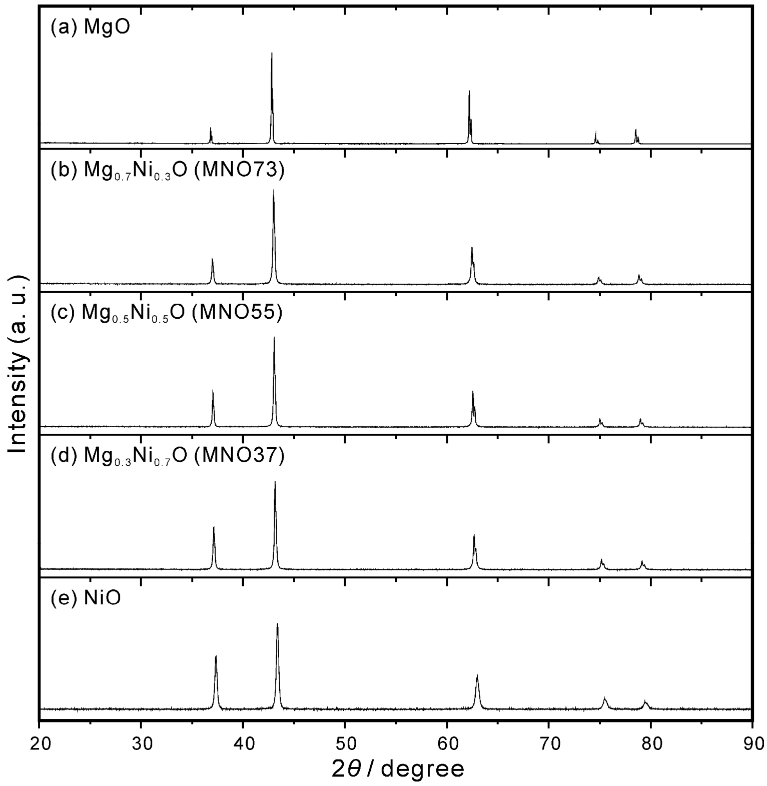

Figure S1. Powder XRD patterns of (**a**) MgO, (**b**) $Mg_{0.7}Ni_{0.3}O$, (**c**) $Mg_{0.5}Ni_{0.5}O$, (**d**) $Mg_{0.3}Ni_{0.7}O$, and (**e**) NiO. The $Mg_{0.7}Ni_{0.3}O$, $Mg_{0.5}Ni_{0.5}O$ and $Mg_{0.3}Ni_{0.7}O$ samples were synthesized by heating at 1300 °C for 10 h in air, and subseqently pulverized by ball-milling for 50 h.

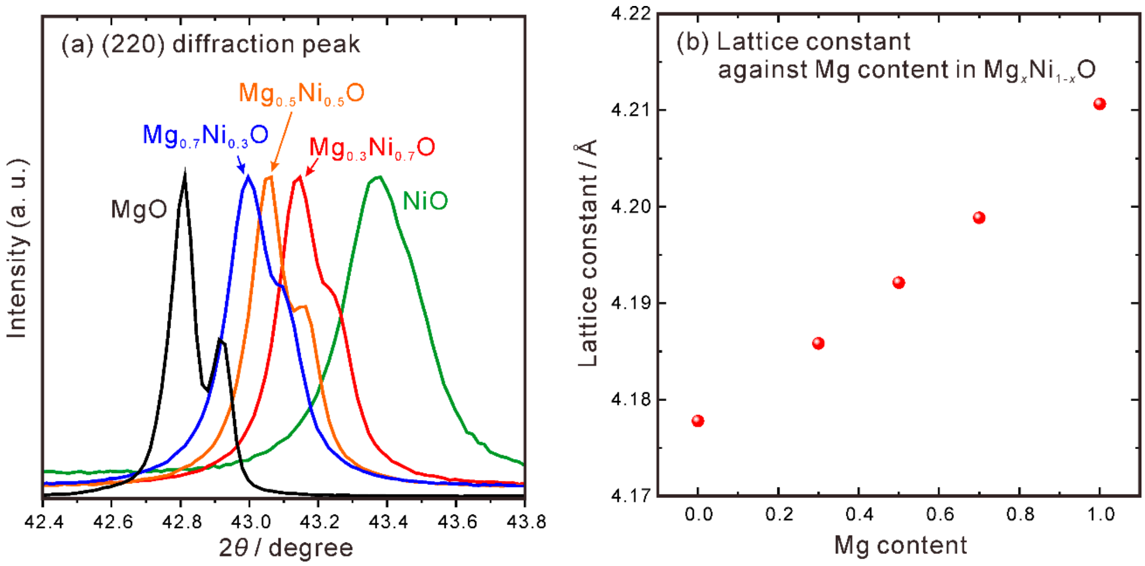

Figure S2. (**a**) Shifting of (220) diffraction peak towards low angle side, and (**b**) increasing lattice constant of the sample with the increasing Mg concentration, indicating that solid solution of MgO-NiO has been prepared successfully, since the six-coordinated radius of Ni(II) (0.690 Å) is smaller than Mg(II) (0.72 Å) [25].

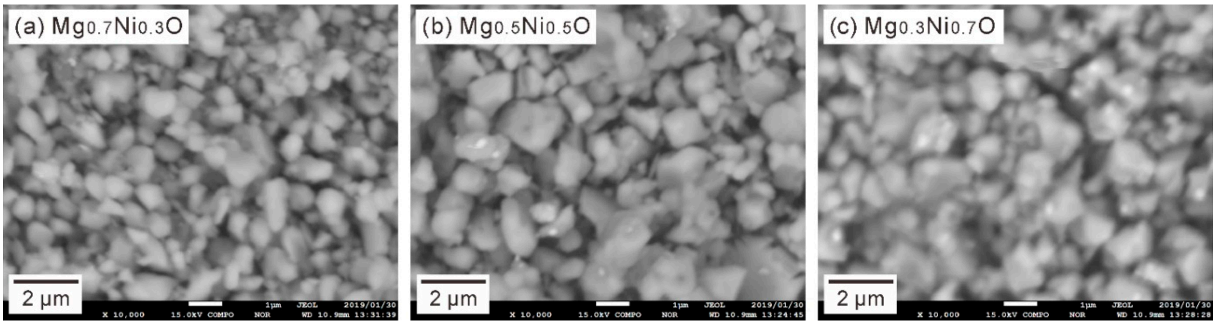

Figure S3. Back scattering electron images of the powders of (**a**) $Mg_{0.7}Ni_{0.3}O$, (**b**) $Mg_{0.5}Ni_{0.5}O$, and (**c**) $Mg_{0.3}Ni_{0.7}O$. The samples were synthesized by heating at 1300 °C for 10 h in air, and subseqently pulverized by ball-milling for 50 h.

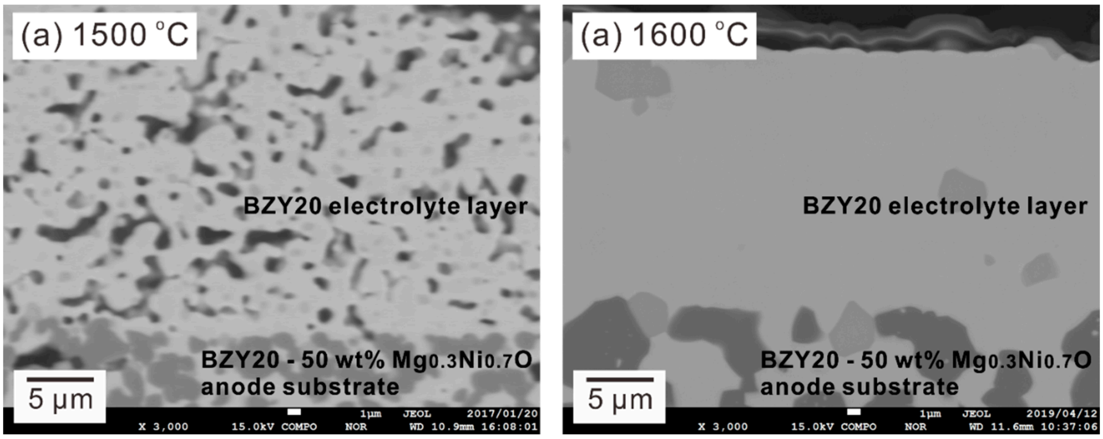

Figure S4. Back scattering electron images of the half cells composed of a BZY20 electrolyte layer and a BZY20-50 wt % $Mg_{0.3}Ni_{0.7}O$ anode substrate, which were co-sintered at (**a**) 1500 °C, and (**b**) 1600 °C in oxygen for 10 h with the open-co-sintering method (Figure 1a).

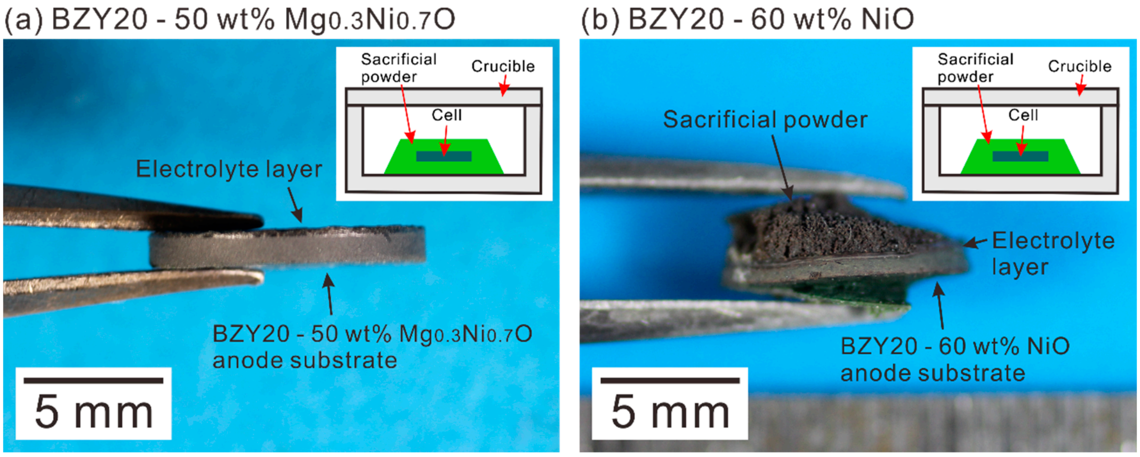

Figure S5. Optical images of the half cells composed of the BZY20 electrolyte layer and (**a**) BZY20-50 wt % $Mg_{0.3}Ni_{0.7}O$, and (**b**) BZY20-60 wt % NiO anode substrates after co-sintering at 1600 °C in oxygen for 10 h with the embedded-co-sintering (Figure 1c) method.

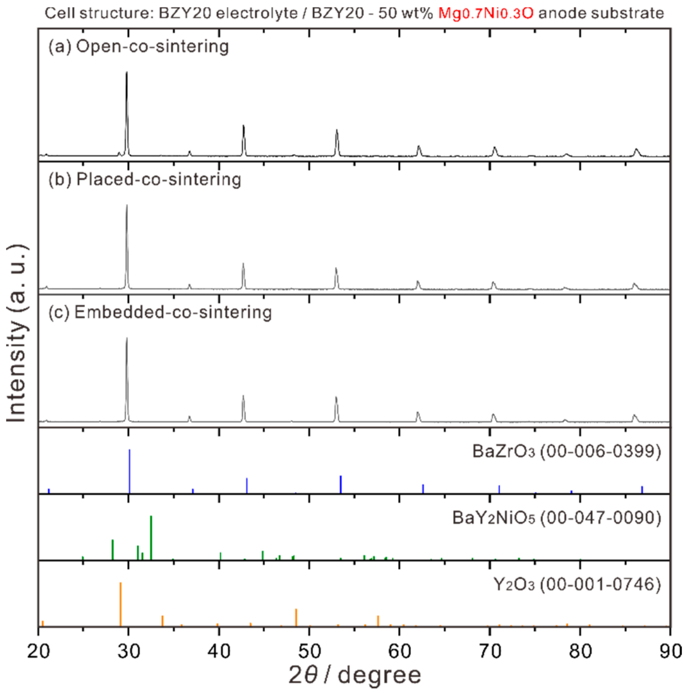

Figure S6. XRD patterns of the electolyte side on the half cells composed of BZY20 electrolyte layers and BZY20-50 wt % $Mg_{0.7}Ni_{0.3}O$ anode substrate co-sintered at 1600 °C in oxygen for 10 h using the (**a**) open-co-sintering, (**b**) placed-co-sintering, and (**c**) embedded-co-sintering methods.

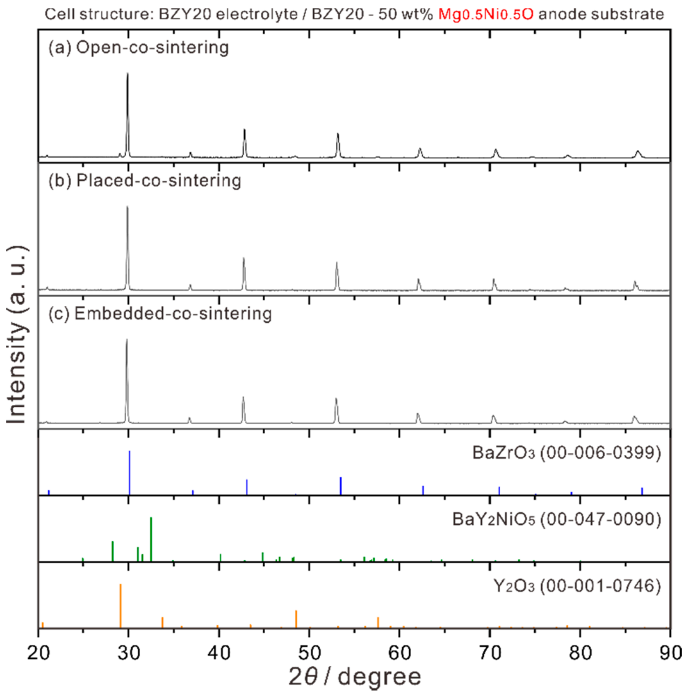

Figure S7. XRD patterns of the electolyte side on the half cells composed of BZY20 electrolyte layers and BZY20-50 wt % $Mg_{0.5}Ni_{0.5}O$ anode substrate co-sintered at 1600 °C in oxygen for 10 h using the (**a**) open-co-sintering, (**b**) placed-co-sintering, and (**c**) embedded-co-sintering methods.

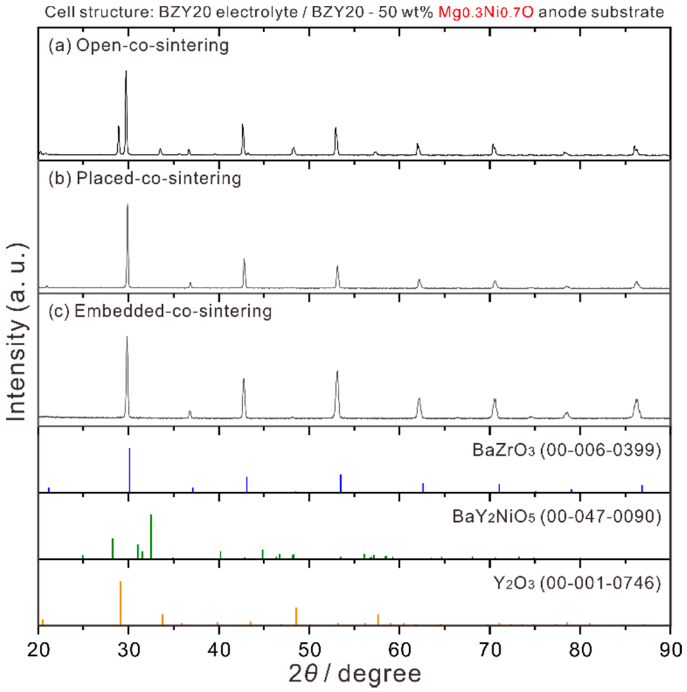

Figure S8 XRD patterns of the electolyte side on the half cells composed of BZY20 electrolyte layers and BZY20-50 wt % $Mg_{0.3}Ni_{0.7}O$ anode substrate co-sintered at 1600 °C in oxygen for 10 h using the (**a**) open-co-sintering, (**b**) placed-co-sintering, and (**c**) embedded-co-sintering methods.

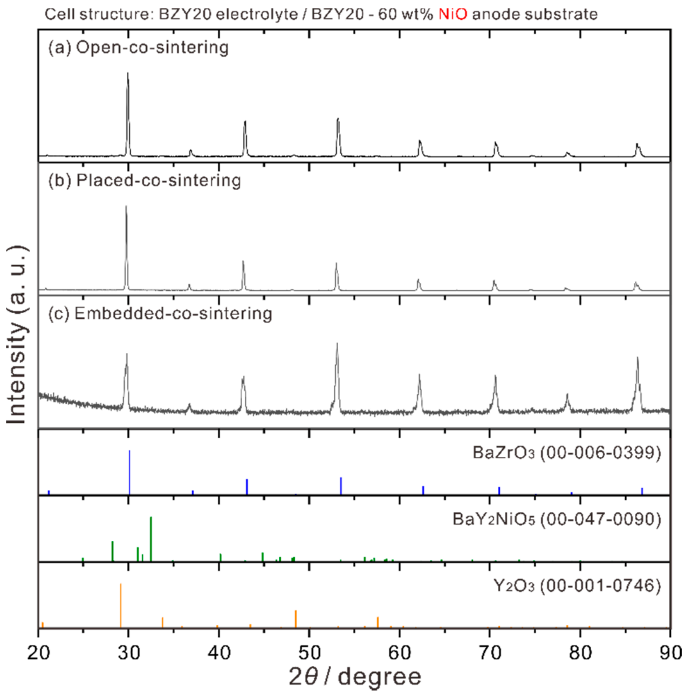

Figure S9. XRD patterns of the electolyte side on the half cells composed of BZY20 electrolyte layers and BZY20-60 wt % NiO anode substrate co-sintered at 1600 °C in oxygen for 10 h using the (**a**) open-co-sintering, (**b**) placed-co-sintering, and (**c**) embedded-co-sintering methods.

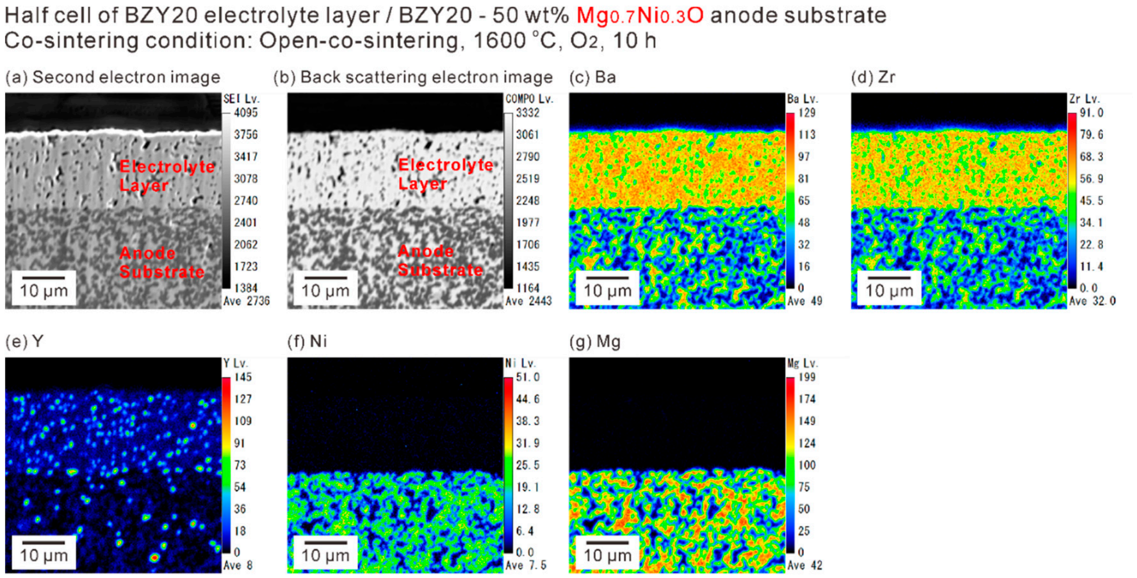

Figure S10. (**a**) The second electron image, and (**b**) back scattering electron image of the cross-section area of the half cell composed of a BZY20 electrolyte and a BZY20-50 wt % $Mg_{0.7}Ni_{0.3}O$ anode substrate, which was co-sintered at 1600 °C in oxygen for 10 h using the open-co-sintering method (Figure 1a). EPMA-WDS elemental mapping analysis was performed to evaluate the distribution of (**c**) Ba, (**d**) Zr, (**e**) Y, (**f**) Ni and (**g**) Mg.

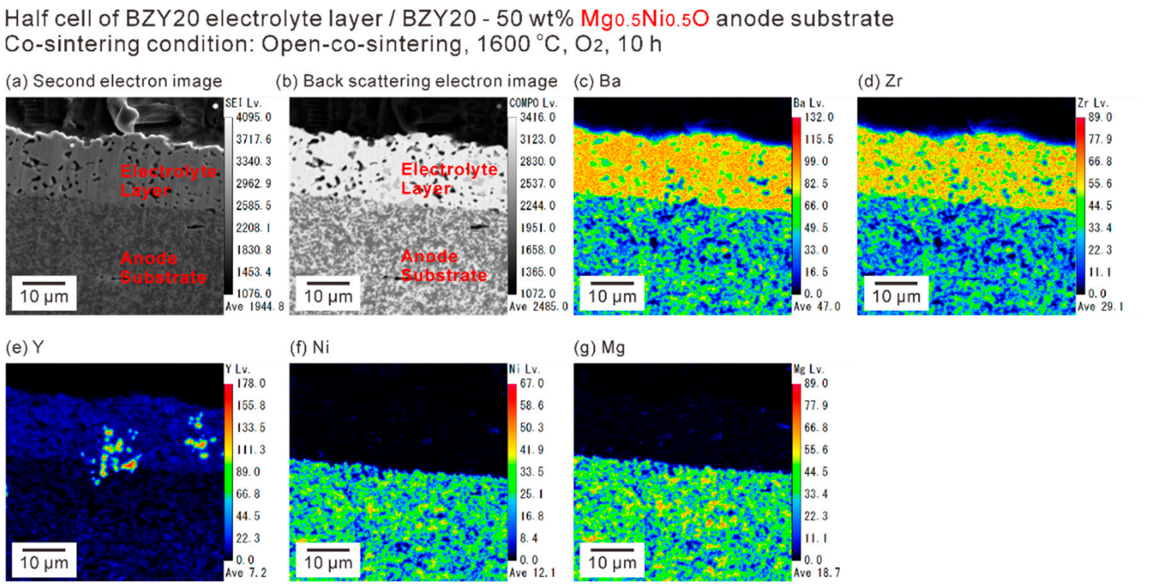

Figure S11. (**a**) The second electron image, and (**b**) back scattering electron image of the cross-section area of the half cell composed of a BZY20 electrolyte and a BZY20-50 wt % $Mg_{0.5}Ni_{0.5}O$ anode substrate, which was co-sintered at 1600 °C in oxygen for 10 h using the open-co-sintering method (Figure 1a). EPMA-WDS elemental mapping analysis was performed to evaluate the distribution of (**c**) Ba, (**d**) Zr, (**e**) Y, (**f**) Ni and (**g**) Mg.

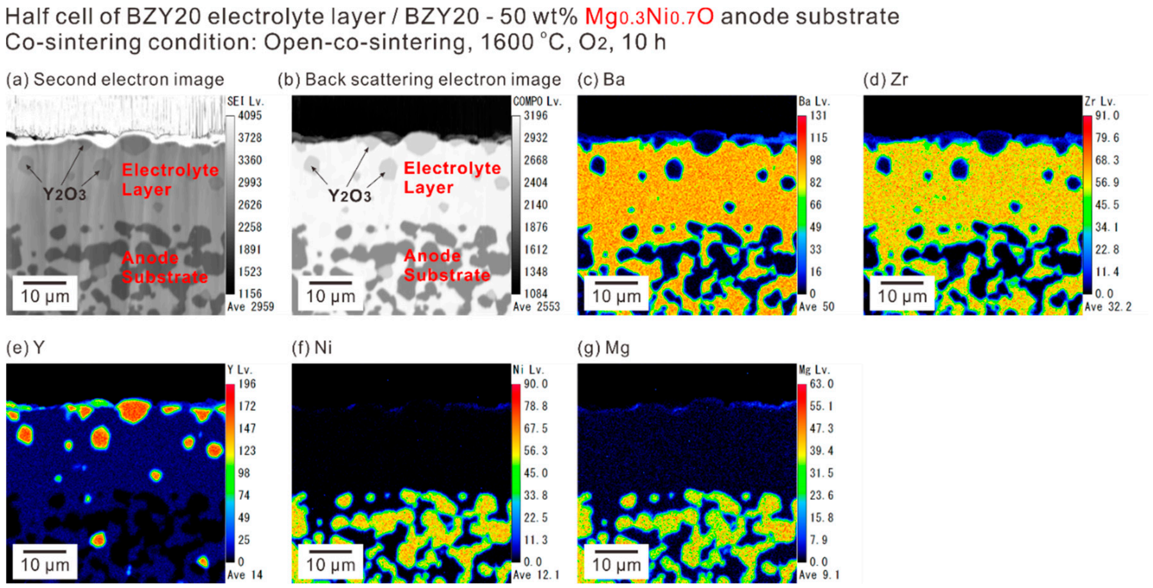

Figure S12. (**a**) The second electron image, and (**b**) back scattering electron image of the cross-section area of the half cell composed of a BZY20 electrolyte and a BZY20-50 wt % $Mg_{0.3}Ni_{0.7}O$ anode substrate, which was co-sintered at 1600 °C in oxygen for 10 h using the open-co-sintering method (Figure 1a). EPMA-WDS elemental mapping analysis was performed to evaluate the distribution of (**c**) Ba, (**d**) Zr, (**e**) Y, (**f**) Ni and (**g**) Mg.

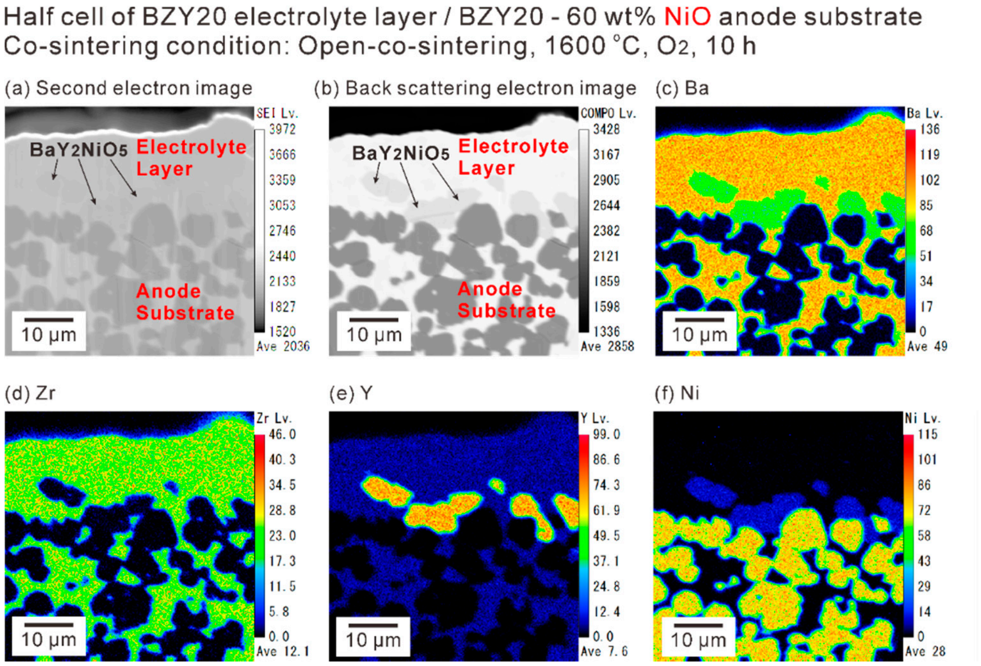

Figure S13. (**a**) The second electron image, and (**b**) back scattering electron image of the cross-section area of the half cell composed of a BZY20 electrolyte and a BZY20-60 wt % NiO anode substrate, which was co-sintered at 1600 °C in oxygen for 10 h using the open-co-sintering method (Figure 1a). EPMA-WDS elemental mapping analysis was performed to evaluate the distribution of (**c**) Ba, (**d**) Zr, (**e**) Y and (**f**) Ni.

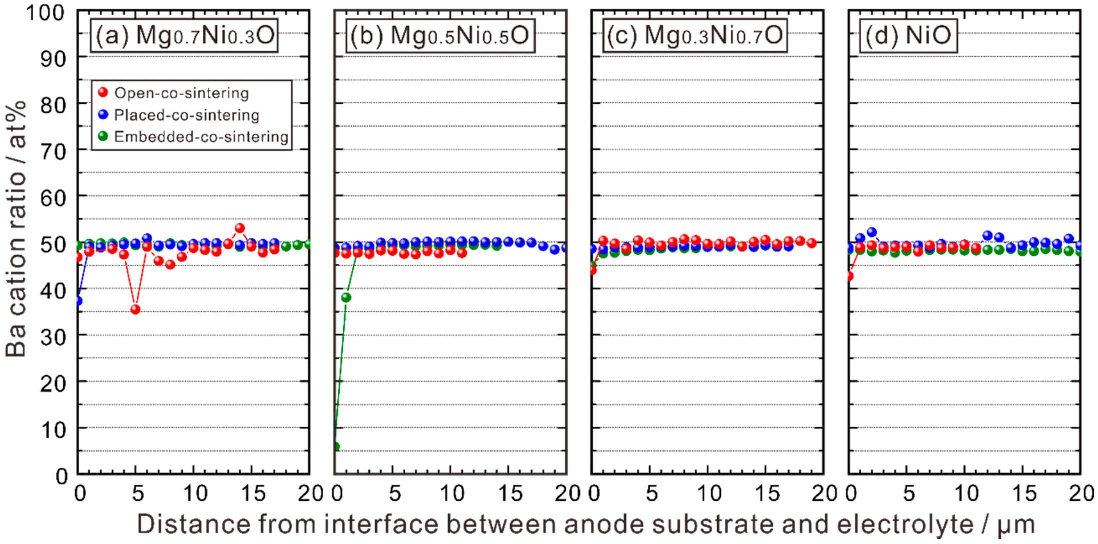

Figure S14. Ba cation ratio in the electrolyte layer of the half cells with the anode substrates of (**a**) BZY20-50 wt % $Mg_{0.7}Ni_{0.3}O$, (**b**) BZY20-50 wt % $Mg_{0.5}Ni_{0.5}O$, (**c**) BZY20-50 wt % $Mg_{0.3}Ni_{0.7}O$, and (**d**) BZY20-50 wt % NiO. All the half cells were co-sintered at 1600 °C in oxygen for 10 h with the method shown in Figure 1. The Ba cation ratio was measured with EPMA-WDS line analysis following the position shown in Figure 2 (red line) from the interface between the anode substrate and the electrolyte.

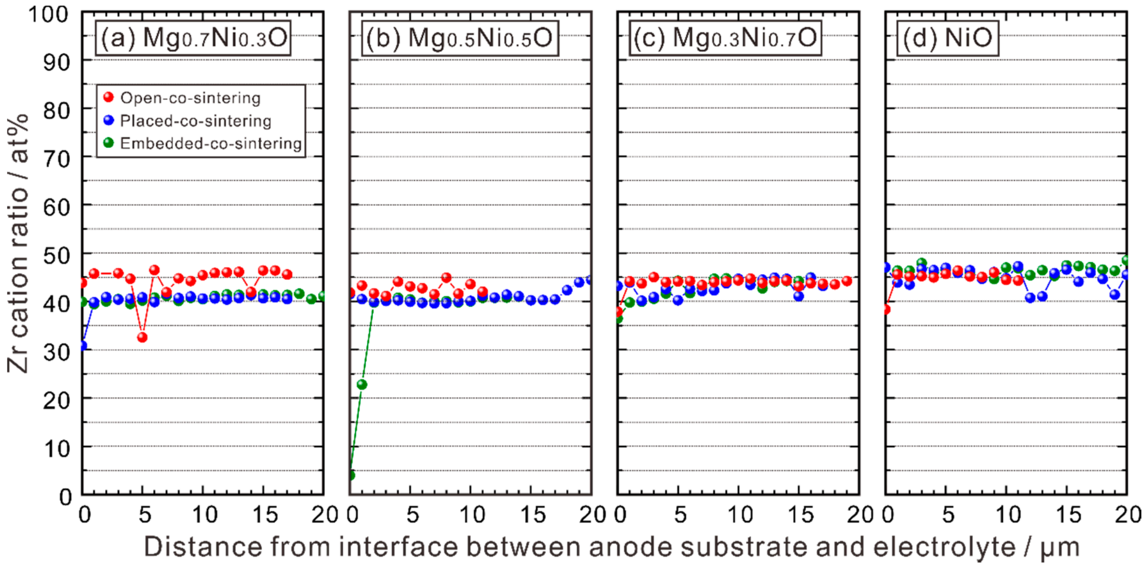

Figure S15. Zr cation ratio in the electrolyte layer of the half cells with the anode substrates of (**a**) BZY20-50 wt % $Mg_{0.7}Ni_{0.3}O$, (**b**) BZY20-50 wt % $Mg_{0.5}Ni_{0.5}O$, (**c**) BZY20-50 wt % $Mg_{0.3}Ni_{0.7}O$, and (**d**) BZY20-50 wt % NiO. All the half cells were co-sintered at 1600 °C in oxygen for 10 h with the method shown in Figure 1. The Zr cation ratio was measured with EPMA-WDS line analysis following the position shown in Figure 2 (red line) from the interface between the anode substrate and the electrolyte.

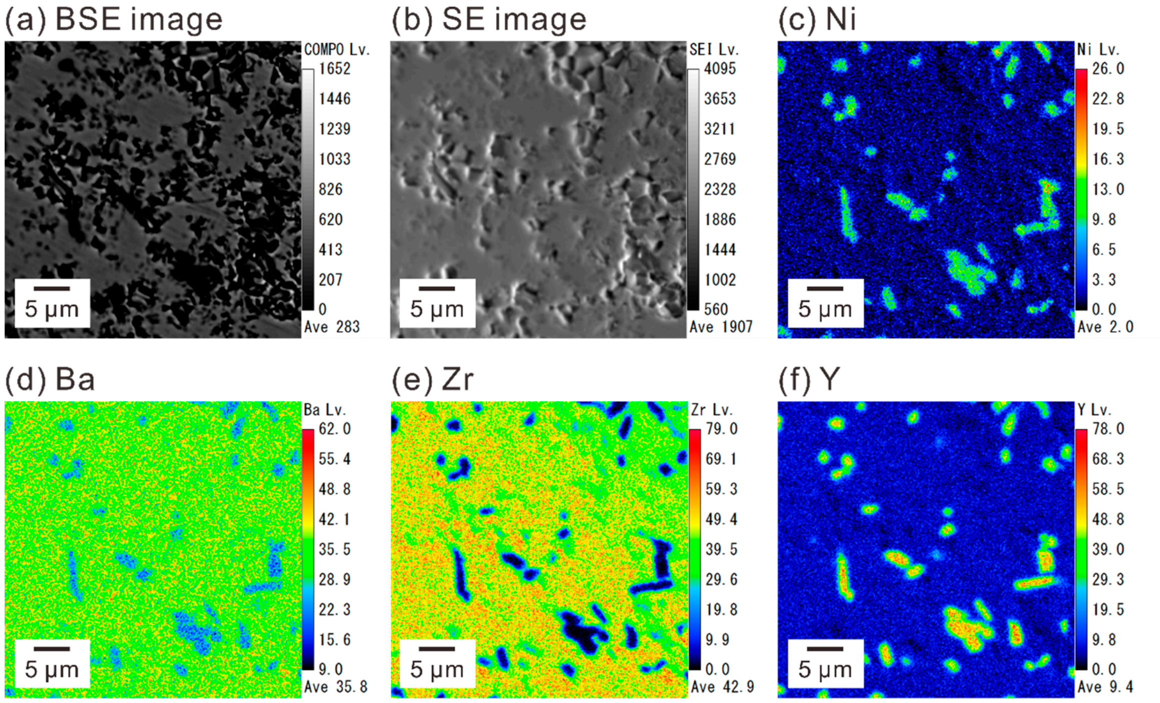

Figure S16. (**a**) Back-scattering electron image and (**b**) second electron image of the cross-section of the $BaZr_{0.78}Y_{0.22}O_{3-\delta}$-2 wt % NiO pellet after sintering at 1500 °C in O_2 for 10 h. EPMA-WDS elemental mapping was performed to see the distribution of (**c**) Ni, (**d**) Ba, (**e**) Zr, and (**f**) Y.

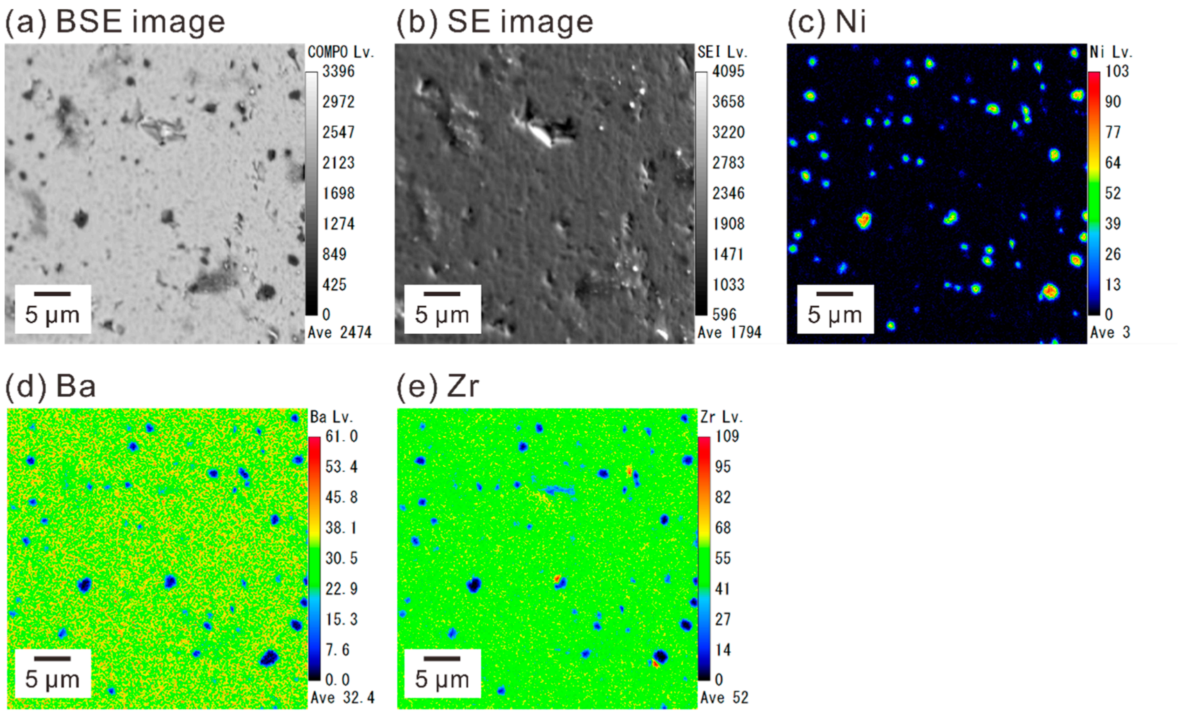

Figure S17. (**a**) Back-scattering electron image and (**b**) second electron image of the cross-section of the $BaZrO_3$-2 wt % NiO pellet after sintering at 1500 °C in O_2 for 10 h. EPMA-WDS elemental mapping was performed to see the distribution of (**c**) Ni, (**d**) Ba, (**e**) Zr, and (**f**) Y.

© 2019 by the authors. Submitted for possible open access publication under the terms and conditions of the Creative Commons Attribution (CC BY) license (http://creativecommons.org/licenses/by/4.0/).
